# Management of donor-specific antibodies in lung transplantation

**DOI:** 10.3389/frtra.2023.1248284

**Published:** 2023-09-29

**Authors:** William Brandon, Colin Dunn, Srinivas Bollineni, John Joerns, Adrian Lawrence, Manish Mohanka, Irina Timofte, Fernando Torres, Vaidehi Kaza

**Affiliations:** ^1^ Department of Internal Medicine, University of Texas Southwestern Medical Center, Dallas, TX, United States; ^2^ Division of Pulmonary and Critical Care Medicine, University of Texas Southwestern Medical Center, Dallas, TX, United States

**Keywords:** donor-specific antibodies, antibody-mediated rejection, lung transplant, desensitization, immunology, donor-derived cell free DNA

## Abstract

The formation of antibodies against donor human leukocyte antigens poses a challenging problem both for donor selection as well as postoperative graft function in lung transplantation. These donor-specific antibodies limit the pool of potential donor organs and are associated with episodes of antibody-mediated rejection, chronic lung allograft dysfunction, and increased mortality. Optimal management strategies for clearance of DSAs are poorly defined and vary greatly by institution; most of the data supporting any particular strategy is limited to small-scale retrospective cohort studies. A typical approach to antibody depletion may involve the use of high-dose steroids, plasma exchange, intravenous immunoglobulin, and possibly other immunomodulators or small-molecule therapies. This review seeks to define the current understanding of the significance of DSAs in lung transplantation and outline the literature supporting strategies for their management.

## 1. Introduction

Rates of lung transplantation have increased considerably over recent years with nearly 34,000 transplants performed between 2010 and 2018, triple the number performed in the 1990s (1). While transplant outcomes have improved marginally over this time period, long-term graft survival been hampered by the frequent occurrence of both acute and chronic rejection. This rejection is driven by both cellular and antibody-mediated processes, the latter of which are thought to be caused primarily by donor specific antibodies (DSAs)—recipient antibodies against foreign donor antigens.

While these antibodies may target any mismatched donor epitope, those of greatest clinical significance in solid organ transplantation are DSAs against HLA antigens. There is a wealth of literature supporting the deleterious effects of anti-HLA DSAs, and even antibodies against non-donor HLA antigens have been shown to adversely affect solid organ allograft outcomes (2). Antibodies against non-HLA donor antigens have been linked to worse transplant outcomes as well, though the clinical significance of their presence is not yet as well defined; antigens previously found to be of particular importance in lung transplantation include the angiotensin type 1 receptor, endothelin type A receptor, and K-α-1-tubulin (3, 4).

The identification and quantification of DSAs is an evolving science. Historically, DSAs were identified using a complement-dependent cytotoxicity assay in which donor lymphocytes are incubated with recipient serum, complement, and a reporter dye to assess the degree of cell death mediated by recipient antibodies against exposed donor antigens. More recently, solid-phase assays have come into use whereby beads coated with HLA antigens are exposed to recipient serum before being tagged with a fluorescent anti-IgG reporter, allowing for the more precise identification of antibodies against specific HLA molecules. The amount of fluorescent signal produced by this method is expressed as the mean fluorescence intensity (MFI), a semi-quantitative measurement allowing for the rough estimation of the presence of and strength of DSAs; of note, the results produced by this method can vary widely depending on the testing methods employed and thresholds set for detection of a positive crossmatch.

As the demand for lung transplantation continues to rise, understanding the impact and management of these DSAs has become paramount. The purpose of this review is to provide a comprehensive overview of the current literature on the clinical implications and management of DSAs in lung transplantation. By synthesizing the existing evidence, this review aims to contribute to the understanding of DSAs' role in lung transplantation and guide clinical practice in optimizing patient outcomes with special attention paid to areas of recent innovation and future direction.

## 2. Clinical implications of donor-specific HLA antibodies

### 2.1. Pre-transplant donor-specific antibodies

Given a historical hesitancy to transplant an HLA haplotype in the presence of a DSA, data regarding outcomes in patients with pre-formed DSAs are sparse. Early studies on outcomes in this population relied on the use of solid-phase assays for the retrospective identification of HLA antibodies that failed to be detected by the complement-dependent cytotoxicity assays in use at the time of transplantation. One such study showed worse 1-year post-transplant survival in patients with pre-formed DSA, with particularly limited survival in those with complement-fixing DSAs or mean fluorescence intensity greater than 5,000 units (5); another identified greater mortality and quicker onset of BOS in patients with class II DSAs, but not in those with only class I (6).

More recently, attempts have been made to transplant in the presence of donor-specific antibodies with or without changes to the induction immunosuppression regimen. One study of 18 patients with pretransplant DSA (but negative complement-dependent cytotoxicity assay) found no difference in survival or time to onset of chronic lung allograft dysfunction (CLAD) when compared to a non-DSA population after a median follow-up period of 1.4 years, however with greater incidence of antibody-mediated rejection (AMR) requiring treatment and greater need for prolonged mechanical ventilation postoperatively (7). Another group trialed transplantation in the presence of low-level [mean fluorescence intensity (MFI) < 6,000 units] DSAs without augmentation of immunosuppression and found no worse survival, time to CLAD, or incidence of AMR after greater than 2-year median followup (8).

### 2.2. De novo donor-specific antibodies

A significant proportion of lung transplant recipients will develop DSAs after transplantation, with one study of 340 patients showing that 47% developed DSAs within the first two years at a median onset of 86 days post-transplant (9). Development of *de novo* DSAs has been linked with adverse transplant outcomes including CLAD and, in some studies, mortality (9–17). In one study, for example, DSA development was associated with a roughly two-fold increase in incidence of CLAD over a median 764 day follow up period (9). Numerous studies support an association between DSA formation and mortality, including two retrospective cohort studies from 2014 (15, 16) and others linking early-onset (within one month from transplant) DSA formation in particular to worse survival (13, 14). More recently, preliminary results from the HALT (HLA Antibodies after Lung Transplantation) study revealed an increased risk of acute cellular rejection after *de novo* DSA formation by prospectively following 119 patients with protocolized DSA surveillance, however the limited follow-up period was likely insufficient to detect differences in other meaningful transplant outcomes (10).

### 2.3. Factors mediating DSA effect

Several characteristics of specific donor-specific antibodies have been found to modulate their effect on transplant outcomes, including their titer, persistence, and propensity to activate the complement system (5, 12, 18). While no well-defined MFI threshold for a clinically significant DSA exists in lung transplantation, one retrospective cohort study found worse 1-year survival (33.3%) in patients with pre-formed DSAs with MFI greater than 5,000 units than in those with detectable but lower-titer DSAs (62.5%–71.4%) (5). Another study found shorter time to CLAD onset in those with *de novo* DSAs at a threshold MFI of only 500 units (13). Antibodies against HLA-DQ antigens appear particularly deleterious as demonstrated in studies showing greater risk for CLAD with anti-DQ DSA when compared to non-DQ DSAs (9, 18). It is otherwise clear that transient DSAs, generally defined as those present on only a single assay, are of less significance than those that are more persistent, as shown in several studies that showed less acute rejection and more CLAD-free and graft survival with only transient DSAs (11, 12, 18). Lastly, DSAs which activate the complement pathway, as demonstrated by positive C1q binding, seem especially harmful when compared to those with C1q-negative DSAs as exhibited in a recent study by Iasella et al. showing more rapid onset of CLAD in this group (18).

### 2.4. Formation of donor-specific antibodies

Considering the adverse transplant outcomes linked to DSA development, it is of considerable interest to identify patients at elevated risk for DSA formation. Retrospective cohort studies have previously identified pre-transplant HLA-DQ mismatch and postoperative platelet transfusion as risk factors for DSA development (12, 17). One study by Kulkarni and colleagues found an independent link between the growth of Pseudomonas aeruginosa, but not other organisms, on respiratory cultures and later development of DSAs, hypothesizing that the particularities of the immune response to Pseudomonas promotes an inflammatory milieu that promotes DSA formation (19). More recently, there has been considerable interest in the use of computer algorithms to estimate the risk of *de novo* DSA development on the basis of pre-transplant epitope mismatch loads (20, 21).

### 2.5. Donor-specific antibodies in the diagnosis of acute rejection

The diagnosis of antibody-mediated rejection of the lung can be challenging to make, and this was historically hindered by an absence of standardized diagnostic criteria. In 2016, the ISHLT published a consensus definition for AMR which requires the following three components in addition to graft dysfunction: presence of DSA, histology suggestive of AMR, and positive C4d staining on biopsy. Patients are classified as having either possible, probable, or definite AMR depending on whether they manifest one, two, or all of these features, respectively (22). C4d staining has come under scrutiny as a meaningful diagnostic requirement, as studies have found this feature to be poorly associated with either the presence of DSAs or other clinical or histologic features of AMR (23–26).

There has been recent interest in the use of donor-derived cell free DNA (ddcfDNA) in the diagnosis of AMR, however this has not yet seen widespread clinical utility. Donor-derived cell free DNA, as a marker of transplant injury, is elevated in cases of both ACR and AMR as well as infectious insults (27–29). One study by Agbor-Enoh and colleagues found the presence of ddcfDNA at a median of 2.8 months before a clinical diagnosis of AMR, even in the absence of spirometric or histologic changes at the time. Cases of AMR in this group were found to have a greater burden of ddcfDNA than in cases of acute cellular rejection (29). While data have been promising so far, more study will be needed to define the clinical utility of this biomarker in the diagnosis and management of rejection.

## 3. Management of donor-specific antibodies in lung transplantation

### 3.1. Management of pre-transplant donor specific antibodies

Given the risks of both acute rejection and CLAD seen after transplantation in the presence of DSAs, numerous strategies for pre-transplant antibody depletion have been investigated. Optimal management of pre-transplant DSAs is poorly defined, and practice varies widely by transplant center; agents trialed include intravenous immunoglobulin (IVIG), plasma exchange (PLEX), monoclonal antibodies, and proteasome inhibitors, with only limited data supporting any particular regimen.

One study demonstrated that peri-operative desensitization in patients with pre-transplant DSAs using a combination of PLEX, IVIG, and anti-thymocyte globulin led to CLAD-free survival comparable to unsensitized patients at a median follow-up of 6.7 years (30); another similar study trialed PLEX, IVIG, anti-thymocyte globulin, and mycophenolic acid and found graft survival and spirometry parameters similar to unsensitized patients at 1 year (31). These data support the efficacy of antibody depletion in the presence of pre-transplant DSAs, however evidence supporting the routine depletion of third-party HLA antibodies preoperatively has been conflicting (32, 33).

Continued research into optimal perioperative anti-HLA antibody management will be essential to expand transplant eligibility to allosensitized patients (34). Of late, one case report showed that imlifidase, an IgG-degrading enzyme derived from Streptococcus pyogenes, was effective in depleting donor-specific antibodies to allow for safe transplant in a sensitized patient (35).

### 3.2. Management of *de novo* DSAs

Most patients are maintained on a three-agent calcineurin inhibitor-based immunosuppression regimen for prevention of both antibody mediated and cellular rejection, however a significant fraction will still develop donor-specific antibodies at some point after transplant. The benefit of treating these *de novo* DSAs in the absence of clinical rejection is uncertain. Several retrospective studies have found at least similar outcomes to unsensitized patients when *de novo* DSAs are treated pre-emptively (36, 37), including a recent study by Keller et al. finding that treatment of clinically silent DSAs was associated with lower risk of CLAD or death (38).

While pre-emptive clearance of DSAs may be helpful, data supporting an optimal antibody depletion regimen are lacking. One study of a combination of PLEX and Rituximab demonstrated efficacy in clearing clinically silent DSAs, but failed to show an improvement in survival or incidence of rejection (39). In a head-to-head comparison of Rituximab and carfilzomib when given for pre-emptive depletion of *de novo* antibodies both agents were found to effectively reduce the MFI of *de novo* DSAs with comparable CLAD-free survival, however Rituximab was associated with less decline in spirometry and a greater duration of DSA clearance (40). Recently, a randomized controlled trial of belatacept, an inhibitor of T-cell signaling agent used in kidney transplantation, as a component of a post-transplant maintenance immunosuppression regimen was stopped early due to increased mortality; no difference in DSA formation was found (41).

### 3.3. Management of AMR

Given the severity of illness associated with antibody-mediated rejection, and particularly hyperacute rejection, there is a strong impetus for antibody depletion in clinical AMR. Outcomes in AMR remain poor despite treatment, with increased risk of progression to CLAD and one study reporting a 26 percent 30-day mortality rate (23, 42–44). There is little consensus on the optimal management of AMR, however most centers rely on a combination of antibody-depleting therapies to include PLEX, IVIG, and Rituximab; a variety of other immunologic and small molecule therapies have been studied in recent years as adjuncts to these as well.

Carfilzomib, for example, was shown in a small observational study to effectively clear C1q + positivity in 10 of 14 patients with AMR when given with IVIG and PLEX, and this improvement was associated with stabilization of FEV1 (45). C5 complement inhibition has been shown to produce favorable effects in mouse models of lung transplantation with anti-C5 therapies being associated with lower rejection scores (46). Ecilizumab in particular has shown clinical promise in kidney transplantation in reducing antibody mediated rejection, however data in lung transplant is limited to case reports (47). Daratumamab has also been explored as an anti-rejection agent in kidney and heart transplant, with data suggesting it could be explored as an option for AMR in lung transplantation (48–50). One recent report compared groups treated for AMR with various antibody-depleting therapies and found that combination regimens including tocilizumab, an anti-IL 6 therapy, had greater clearance of DSAs and graft survival. Lastly, exploration of regulatory T-cells and bronchus-associated lymphoid tissue has highlighted the ability for local T-cells to reduce B-cell activity in allografts and may be a future therapeutic target (51).

As summarized above, there are only limited data supporting the choice of any specific antibody depletion regimen in lung transplantation; a selection of some of the available evidence for each is presented in Table 1.

**Table 1:** Selection of evidence for specific antibody-depletion regimens in lung transplantation.

First author, year of publication	Journal	Clinical scenario	Therapy	Outcome	Limitations	Reference
January, 2023	The Journal of Heart and Lung Transplantation	Total of 27 LTRs with AMR	9 patients treated with various regimens including tocilizumab vs 18 patients treated without the use of tocilizumab	Tocilizumab-containing regimens were associated with greater clearance of DSAs, lower recurrence of DSAs, greater retransplantation-free survival.	Medication regimens were not standardized, observational	(52)
Pham, 2021	Transplantation Direct	31 episodes of AMR	IVIG, PLEX, and carfilzomib	82.1% of episodes had positive response: 1+ of clearance of DSA, decline in MFI by >3,000, or loss of C1q fixation.	Observational study	(53)
Muller, 2018	Transplantation	Single patient with AMR 1 week after transplant with positive class I and II DSAs	Steroids, IVIG, Rituximab, and eculizumab	Rapid clinical improvement with at least 3-year CLAD-free survival	Single observation	(47)
Vacha, 2017	Clinical Transplantation	16 LTRs with DSAs and accompanying graft dysfunction	PLEX, steroids, bortezomib, Rituximab, and IVIG	69% survival to 6 months; among survivors, 27% cleared all DSAs, 36% had preserved graft function at 6 months.	Observational study	(54)
Ius, 2016	Transplantation	113 LTRs with early DSAs (seen before index hospitalization discharge) without accompanying graft dysfunction	Either IVIG (IgM-enriched)/Rituximab (Group A) or PLEX/Rituximab (Group B)	DSA clearance by end of therapy in 92% vs 64% (*p* = .002) of patients between groups A and B. Overall survival was better in group A.	Observational study	(55)
Otani, 2014	Transplant Immunology	9 LTRs with clinical AMR, defined as DSAs with MFI >5,000 and unexplained drop in FEV1, onset within 12 months of transplant	High-dose IV steroids, PLEX, IVIG, and Rituximab	Median MFI declined from 5,292 to >2,409. 5 recovered rapidly, the remaining 4 died from progression of pre-existing CLAD	Observational study	(43)
Daoud, 2013	Transplant Immunology	14 LTRs with at least one feature of AMR (linear C4d staining, pulmonary capillaritis, or DSAs)	PLEX/IVIG, with or without Rituximab	Of those treated, 4/7 cleared DSAs, 5/7 remained alive at 803-day follow-up.	Many subjects had coexisting infection, observational study	(56)
Stuckey, 2012	Annals of Pharmacotherapy	Single LTR transplanted across HLA mismatch complicated by early graft dysfunction with class I and II DSAs	PLEX, IVIG, and Bortezomib	Clearance of DSAs by day 255 and preserved graft function at 2 years post-transplant	Single observation	(57)
Dawson, 2012	The Journal of Heart and Lung Transplantation	Single highly sensitized LTR who developed hyperacute rejection	PLEX, IVIG, Rituximab, eculizumab, and bortezomib	Successfully weaned from ECMO, ventilatory support, and hemodialysis with CLAD-free survival to at least one year	Single observation	(58)
Hachem, 2010	The Journal of Heart and Lung Transplantation	65 patients who developed *de novo* DSAs	Either IVIG/Rituximab or IVIG alone	Patients treated with antibody-depletion therapy had similar incidence of acute rejection, lymphocytic bronchiolitis, and BOS as those without DSAs. Depletion of DSAs was associated with better survival than in persistent DSAs.	Absence of strict control group	(36)

LTRs, lung transplant recipients.

## 4. Conclusion

The development of antibodies to donor HLA antigens is a challenging problem in lung transplantation both for donor selection as well as for long-term graft function. There is a wealth of literature supporting the deleterious effects of donor-specific antibodies pre- and post-operatively, however high-quality data supporting the indications for DSA clearance and optimal treatment regimens are lacking. Areas of active research interest include the use of ddcfDNA for the identification of graft injury and computational models for the prediction of post-operative DSA development. Given the critical need for donor lungs and disappointing long-term graft function outcomes, further investigation into optimal management of DSAs will be essential both to expand the donor pool and produce more durable graft function post-operatively.
